# Durability of glycemic control with insulin lispro mix 75/25 versus insulin glargine for older patients with type 2 diabetes

**DOI:** 10.1007/s40520-013-0140-8

**Published:** 2013-10-03

**Authors:** Lois Jovanovič, Anne L. Peters, Honghua H. Jiang, Dana S. Hardin

**Affiliations:** 1 Sansum Diabetes Research Institute, Santa Barbara, CA USA; 2 University of Southern California Keck School of Medicine, Los Angeles, CA USA; 3 Eli Lilly and Company, Lilly Corporate Center, Drop Code 223, Indianapolis, IN 46285 USA

**Keywords:** Type 2 diabetes mellitus, Insulin, Hemoglobin A_1c_, Aged

## Abstract

**Background and Aims:**

Few studies have evaluated long-term durability of glycemic control in older patients. The aim of this study was to compare durability of glycemic control of twice-daily insulin lispro mix 75/25 (LM75/25; 75 % insulin lispro protamine suspension, 25 % insulin lispro) and once-daily insulin glargine (GL) added to oral antihyperglycemic medications in older patients (≥65 years of age).

**Methods:**

Patients were participants in the maintenance phase of the DURABLE trial. During the initiation phase, patients with type 2 diabetes were randomized to LM75/25 or GL. After 6 months, patients with hemoglobin A_1c_ (HbA_1c_) ≤7.0 % advanced to the 24-month maintenance phase. The primary objective was between-group comparison of duration of maintaining the HbA_1c_ goal in older patients (≥65 years of age). A similar analysis was conducted for older patients achieving HbA_1c_ ≤6.5 % in the initiation phase.

**Results:**

Median time of maintaining HbA_1c_ goal was longer in LM75/25 versus GL (19.6 versus 15.4 months, *p* = 0.007) and more LM75/25 patients maintained goal versus GL (49.2 versus 30.4 %; *p* = 0.003). HbA_1c_ reduction from baseline was greater in LM75/25 versus GL (−1.56 ± 0.10 versus −1.24 ± 0.11 %; *p* = 0.003). Post-meal glucose was significantly lower in LM75/25 versus GL (158.86 ± 3.42 versus 171.67 ± 4.51 mg/dL; *p* = 0.017). No differences were observed in overall and severe hypoglycemia. LM75/25 patients had higher daily insulin doses (0.41 ± 0.02 versus 0.32 ± 0.02 units/kg/day; *p* < 0.001) and more weight gain (5.47 ± 0.49 versus 3.10 ± 0.53 kg; *p* = 0.001). Similar results were generally obtained in older patients with HbA_1c_ ≤6.5 %.

**Conclusions:**

In our evaluation of older patients from a larger trial, LM75/25 appeared to provide longer durability of glycemic control, as well as a greater number of patients maintaining HbA_1c_ goal versus GL.

## Introduction

Type 2 diabetes is one of the most common chronic conditions of older age (≥65 years). It is estimated that 20 % of the older population suffer from type 2 diabetes [1, 2] and that type 2 diabetes is associated with greater morbidity and mortality in older patients [2]. Management of type 2 diabetes in older patients can be more challenging and problematic than in younger patients [3]. Compared with younger patients, older patients are more likely to have comorbid conditions that may complicate management.

There is increased interest in the efficacy and safety of treatment regimens in older patients, particularly in understanding safe hemoglobin A_1c_ (HbA_1c_) targets. To date, few studies have evaluated long-term durability of glycemic control in older patients, particularly in the case of insulin therapy.

The DURABLE trial (assessing the DURAbility of Basal versus Lispro mix 25 insulin Efficacy) was designed to study the efficacy, safety, and durability of two starter insulin regimens (twice-daily insulin lispro mix 75/25 [LM75/25, 75 % insulin lispro protamine suspension, 25 % insulin lispro] versus once-daily insulin glargine [GL]) in a large, diverse cohort of patients with type 2 diabetes [4, 5]. At the completion of the 24-week initiation phase, with continuation of pre-study oral anti-hyperglycemic medications (OAMs), in the overall study population (30–80 years of age), efficacy was slightly greater in the LM75/25 versus GL group, with greater overall prevalence of hypoglycemia but less nocturnal hypoglycemia [6]. Patients with HbA_1c_ ≤7.0 % continued into a 24-month maintenance phase evaluating how long each insulin regimen could maintain HbA_1c_ goal. At completion of the maintenance phase, a modestly longer duration of glycemic control was achieved in the LM75/25 versus GL group in the overall group [7]. Wolfenbuttel et al. [8] previously reported a post-hoc analysis of efficacy and safety from the initiation phase of the DURABLE trial of a subgroup of patients ≥65 years of age and found that LM75/25 demonstrated a lower endpoint HbA_1c_ and a higher percentage of patients reaching HbA_1c_ target of <7.0 %, but with more weight gain and higher rates of hypoglycemia compared with GL.

The objective of the present analysis was to compare the durability of glycemic control of LM75/25 versus GL in the subgroup of older patients (≥65 years of age) participating in the maintenance phase of the DURABLE Trial.

## Methods

### Study design

This study was a post-hoc analysis of a subset of data from the DURABLE Trial maintenance phase. A detailed description of the DURABLE study design has been previously published [4]. Briefly, the DURABLE trial was a randomized, open-label, parallel, 30-month trial conducted in 11 countries. The trial enrolled insulin-naïve patients with type 2 diabetes, aged ≥30 to <80 yrs, with HbA_1c_ >7.0 %, on at least 2 oral OAMs: ≥1,500 mg/day metformin (MET); at least ½ maximal daily dose sulfonylurea (SU), or thiazolidinedione (TZD [≥30 mg/day pioglitazone or ≥4 mg/day rosiglitazone]). In the 24-week initiation phase [6], patients were randomized 1:1 to LM75/25 twice daily or GL once daily, both in combination with pre-study OAM. After the 24-week initiation phase, patients with HbA_1c_ ≤7.0 % were followed for up to an additional 24 months (maintenance phase [7]) to evaluate how long HbA_1c_ goal could be maintained. The HbA_1c_ goal was either HbA_1c_ ≤7.0 % or HbA_1c_ >7.0 %, but increased <0.4 % from last HbA_1c_ ≤7.0 %. The LM75/25 starting dose was ten units twice daily, and the GL starting dose was ten units once daily, both added to pre-study OAMs. Insulin was adjusted to achieve HbA_1c_ ≤6.5 % using regimen-specific insulin-titration algorithms based on self-monitored plasma glucose review [6]. Patients monitored plasma glucose at least twice daily (before morning and evening meals). During the 6-month initiation phase, dose adjustments were reviewed by an external data monitoring committee; this was not continued during maintenance because patients had an HbA_1c_ ≤7.0 %. Doses were assessed and adjusted ≤3 months according to patients’ twice-daily self-monitored plasma glucose values. The maintenance phase did not include rescue therapy; therefore, patients were discontinued from the trial if HbA_1c_ increased to >7.5 %. Hypoglycemia was recorded any time a patient experienced symptoms of hypoglycemia or had a self-monitored plasma glucose ≤70 mg/dL, and the event was deemed severe if the patient required assistance.

The present analysis only included data from older patients (≥65 years of age) and primarily compared duration of maintaining HbA_1c_ goal (HbA_1c_ ≤7.0 % or HbA_1c_ >7.0 % with <0.4 % increase from last HbA_1c_ ≤7.0 %) for LM75/25 versus GL. Analyses were also conducted for older patients (≥65 years of age) achieving 24-week HbA_1c_ targets ≤6.5 %. Secondary analyses included HbA_1c_ change from baseline (randomization), plasma glucose profiles at endpoint, weight change, total daily insulin dose, hypoglycemia rate, and incidence.

### Statistical methods

All analyses were conducted based on the intent-to-treatment population. The primary efficacy measure, the time of maintaining glycemic control, was compared between treatment groups with a stratified log-rank test controlling for country, TZD, and SU use. Categorical variables were compared with the Fisher exact test. HbA_1c_ change from baseline to endpoint, endpoint insulin dose, weight change, and 7-point self-monitored plasma glucose profile were compared using analysis of covariance (ANCOVA). Treatment, baseline value (if applicable), and stratification variables (country, TZD use, and SU use) were included in the model. Hypoglycemia rates were compared using the negative binomial model with factors of treatment, country, TZD use, and SU use. All analyses were conducted using SAS 9.2 (SAS Institute Inc., Cary, NC).

## Results

### Patient disposition and baseline characteristics

**Fig. 1:**
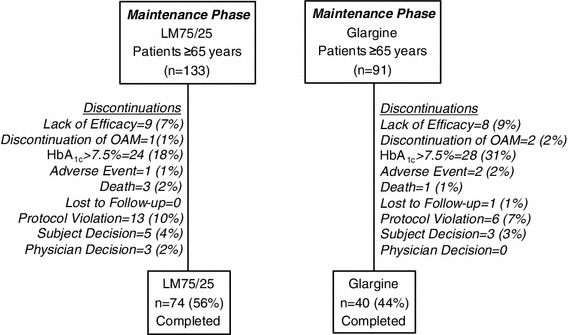
Patient disposition in the maintenance phase of the DURABLE trial for patients ≥65 years. *Glargine* insulin glargine, *LM75/25* insulin lispro mix 75/25 (75 % insulin lispro protamine suspension, 25 % insulin lispro), *OAM* oral anti-hyperglycemic medication

Of the 892 patients who entered the maintenance phase, 224 were older (≥65 years of age) (LM75/25, *n* = 133; GL, *n* = 91) and made up the study population for this post-hoc analysis (Fig. 1). A portion of these older patients achieved the 24-week HbA_1C_ target ≤6.5 % (LM75/25, *n* = 72; GL, *n* = 39). In all older patients, baseline demographic characteristics were similar between treatment groups with the exception of use of the SU/TZD combination which was lower in the LM75/25 group compared with the GL group (6.1 versus 13.5 %, *p* = 0.026) (Table 1). In older patients achieving 24-week HbA_1C_ targets ≤6.5 % (LM75/25, *n* = 72; GL, *n* = 39) all baseline demographic characteristics were similar between the LM75/25 and GL subgroups (Table 1). Baseline glycemic control (HbA_1C_) was similar between treatment groups at study entry in patients ≥65 years of age (LM75/25 8.5 % versus GL 8.4 %) and in patients ≥65 years of age achieving 24-week HbA_1C_ targets ≤6.5 % (LM75/25 8.3 % versus GL 8.2 %).

**Table 1:** Baseline demographic characteristics

Characteristic	All	HbA_1C_ ≤6.5 %^a^	All	HbA_1C_ ≤6.5 %^a^
	Glargine *n* = 91^b^	Glargine *n* = 39^c^	LM75/25 *n* = 133^d^	LM75/25 *n* = 72^e^
Age, years	69.6 ± 4.0	69.4 ± 4.0	69.5 ± 4.0	69.4 ± 4.0
Sex (male/female)	55/36	25/14	79/54	39/33
Race/ethnicity, *n* (%)				
Caucasian	81 (89.0)	34 (87.2)	112 (84.2)	62 (86.1)
African descent	4 (4.4)	2 (5.1)	8 (6.0)	3 (4.2)
Asian	2 (2.2)	0	3 (2.3)	0
Hispanic	4 (4.4)	3 (7.7)	8 (6.0)	6 (8.3)
Other	0	0	2 (1.5)	1 (1.4)
Weight, kg	90.5 ± 18.2	90.5 ± 19.5	88.8 ± 18.4	90.8 ± 17.6
BMI, kg/m^2^	32.5 ± 5.4	32.3 ± 5.2	31.6 ± 5.2	32.5 ± 5.2
Diabetes duration, years	11.0 ± 6.5	9.7 ± 5.3	12.3 ± 8.0	11.3 ± 7.0
HbA_1C_ (%)	8.4 ± 0.9	8.2 ± 0.7	8.5 ± 1.1	8.3 ± 1.0
FBG				
(mg/dL)	183.1 ± 46.3	186.9 ± 47.5	176.6 ± 47.6	172.3 ± 47.7
(mmol/L)	10.2 ± 2.6	10.4 ± 2.6	9.8 ± 2.6	9.6 ± 2.7
Concomitant OAMs, *n* (%)				
Patients with 3 drugs	15 (16.9)	4 (10.3)	32 (24.2)	16 (22.5)
Patients with 2 drugs	74 (83.1)	35 (89.7)	100 (75.8)	55 (77.5)
Sulphonylurea/TZD	12 (13.5)	4 (10.3)	8 (6.1)^f^	5 (7.0)
Sulphonylurea/metformin	59 (66.3)	30 (76.9)	82 (62.1)	42 (59.2)
TZD/metformin	3 (3.4)	1 (2.6)	10 (7.6)	8 (11.3)

Data are mean ± SD, unless otherwise indicated

*BMI* body mass index, *FPG* fasting plasma glucose, *HbA*_*1C*_ hemoglobin A_1c_, *Glargine* insulin glargine, *LM75/25* insulin lispro mix 75/25 (75 % insulin lispro protamine suspension, 25 % insulin lispro), *OAM* oral anti-hyperglycemic medication, *TZD* thiazolidinedione

^**a**^Patients with HbA_1C_ ≤6.5 % at the end of the 24-week initiation phase

^b^Except for HbA_1C_ (*n* = 89), FPG (*n* = 86) and Concomitant OAMs (*n* = 89)

^c^Except for HbA_1C_ (*n* = 37) and FPG (*n* = 36)

^d^Except for HbA_1C_ (*n* = 131), FPG (*n* = 130) and Concomitant OAMs (n = 132)

^e^Except for HbA_1C_ (n = 71), FPG (n = 69) and Concomitant OAMs (*n* = 71)

^f^ *p* = 0.026 versus Glargine (All)

### Glycemic control

**Fig. 2:**
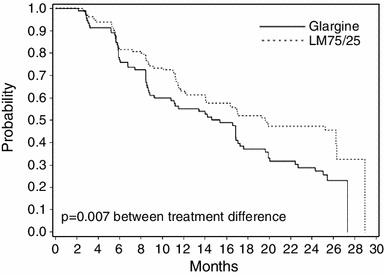
Time to failure to maintain HbA_1C_ goal. *Glargine* insulin glargine, *LM75/25* insulin lispro mix 75/25 (75 % insulin lispro protamine suspension, 25 % insulin lispro)

**Fig. 3:**
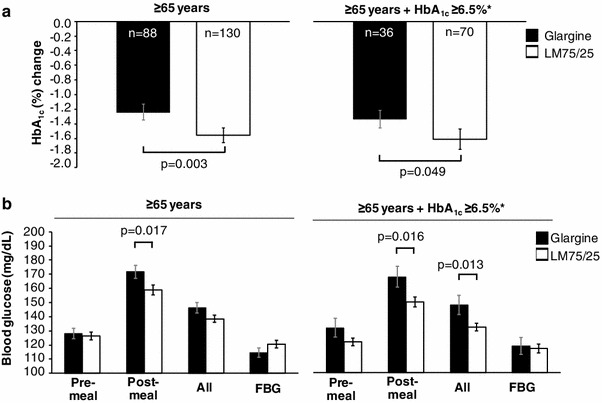
**a** HbA_1C_ change from baseline to endpoint and **b** Plasma glucose at endpoint. *HbA*_*1C*_ glycosylated hemoglobin A1C, *FPG* fasting plasma glucose, *Glargine* insulin glargine, *LM75/25* insulin lispro mix 75/25 (75 % insulin lispro protamine suspension, 25 % insulin lispro). Data are mean ± SEM. *Patients with HbA_1C_ ≤6.5 % at the end of the 24-week initiation phase

The median time of maintaining HbA_1C_ goal was longer in LM75/25 versus GL (19.6 months [95 % CI = 14.0, 26.3] versus 15.4 months [95 % CI = 9.2, 17.3]; *p* = 0.007, Fig. 2). More patients in LM75/25 maintained HbA_1C_ goal versus GL (49.2 versus 30.4 %; *p* = 0.003). HbA_1C_ reduction from baseline was greater in LM75/25 versus GL (−1.56 ± 0.10 versus −1.24 ± 0.11 %; *p* = 0.003) (Fig. 3a). Endpoint fasting blood glucose was similar in LM75/25 versus GL, but post-meal glucose was significantly lower in LM75/25 versus GL (158.86 ± 3.42 versus 171.67 ± 4.51 mg/dL; *p* = 0.017).

Analysis of older patients who achieved HbA_1C_ ≤6.5 % also revealed a greater HbA_1C_ reduction from baseline in LM75/25 versus GL (−1.61 ± 0.14 versus −1.34 ± 0.12 %; *p* = 0.049) (Fig. 3a). Additionally, post-meal glucose and the mean of all blood glucose measurements were significantly lower in LM75/25 versus GL (150.02 ± 3.52 versus 167.67 ± 7.36 mg/dL; *p* = 0.016) and (132.14 ± 2.69 versus 147.83 ± 6.62 mg/dL; *p* = 0.013), respectively (Fig. 3b).

### Body weight and insulin dose

At endpoint, patients ≥65 years of age treated with LM75/25 gained more weight than did patients treated with GL (5.47 ± 0.49 versus 3.10 ± 0.53 kg; *p* = 0.001). Similar weight gains were also observed for the two treatment groups (LM75/25, *n* = 71; 5.02 ± 5.69 kg versus GL, *n* = 38, 2.89 ± 5.44 kg, *p* = 0.159) in patients ≥ 65 years of age achieving 24-week HbA_1C_ targets ≤6.5 %. The total daily insulin dose at endpoint was higher in patients ≥65 years of age treated with LM75/25 versus GL (0.41 ± 0.02 versus 0.32 ± 0.02 units/kg/day; *p* < 0.001) but similar for LM75/25 versus GL in patients ≥65 years of age achieving 24-week HbA_1C_ targets ≤6.5 %.

### Hypoglycemia

At endpoint, the rate (mean ± SD) of overall hypoglycemia was 23.21 ± 37.28 versus 19.25 ± 29.34 episodes/patient/year, *p* = 0.669. The rate of nocturnal hypoglycemia was 8.74 ± 21.30 versus 10.99 ± 20.64 episodes/patient/year, *p* = 0.919) and incidence of severe hypoglycemia (2.3 versus 3.3 %, *p* = 0.436). Thus hypoglycemia was similar for LM75/25 versus GL in patients ≥65 years. Interestingly, in patients ≥65 years of age achieving 24-week HbA_1C_ targets ≤6.5 %, the rate (mean ± SD) of overall hypoglycemia (22.43 ± 39.91 versus 18.52 ± 24.58 episodes/patient/year, *p* = 0.604) and incidence of severe hypoglycemia (1.4 versus 5.3 %, *p* = 0.212) were similar for LM75/25 versus GL.

## Discussion

This post-hoc analysis represents the first comparison of GL and premix analog insulin for maintaining long-term insulin therapy in a large population of older patients (≥65 years of age) with type 2 diabetes. LM75/25 therapy resulted in a longer durability of glycemic control, but with more weight gain which was associated with modestly higher daily insulin doses. Hypoglycemia rates and incidence of severe hypoglycemia were similar for LM75/25 versus GL. A greater number of patients treated with LM75/25 maintained HbA_1C_ goal compared with patients treated with GL.

Reaching and maintaining glycemic targets reduces the risk of long-term complications in diabetes. When evaluating therapies, it is important to examine the glycemic durability (the length of time a patient is able to maintain glycemic control). In older patients, LM75/25 therapy resulted in a longer durability of glycemic control, and more patients maintained HbA_1C_ goal when compared with GL therapy. The findings of the current analysis are also comparable to the results of the maintenance phase of the overall DURABLE trial (ages 30–80 years) in which LM75/25 therapy resulted in a longer durability of glycemic control and was associated with more weight gain and modestly higher daily insulin doses [7]. Similarly, a greater number of LM75/25-treated patients maintained HbA_1C_ goal compared with patients treated with GL, and no differences were observed in hypoglycemia.

Clinical studies have shown that postprandial glucose is an important contributor to overall glycemic control, particularly as HbA_1C_ values approach lower target values [9]. In addition, targeting postprandial glucose may reduce the risk for many diabetes-related complications, but this is still a subject of intense debate [10]. Premixed insulin analogs address both preprandial and postprandial blood glucose targets to more closely mimic physiological insulin secretion [11]. In the present study, LM75/25 demonstrated better postprandial glycemic control compared with GL in all older patients.

Fear of hypoglycemia remains one of the key barriers to initiating and optimizing insulin therapy [12, 13]. Successful insulin therapy involves a delicate balance between achieving adequate glycemic control while preventing hypoglycemia. Considering the greater risk for developing hypoglycemia (and severe hypoglycemia) in older patients [3], and the greater morbidity associated with hypoglycemia in this population [14], an important finding in this study was similar rates of overall hypoglycemia, nocturnal hypoglycemia, and the incidence of severe hypoglycemia between LM75/25 compared with GL in older patients.

In the subgroup of older patients achieving the more stringent HbA_1C_ target of ≤6.5 %, LM75/25 also demonstrated better postprandial glycemic control compared with GL and in addition, overall plasma glucose at endpoint was lower in LM75/25 versus GL in older patients with HbA_1C_ ≤6.5 %. Similarly, the rates of hypoglycemia were similar between the treatment groups in this subgroup of older patients that achieved HbA_1C_ ≤6.5 %, although the numbers in this subgroup are relatively small.

These findings contrast with the earlier analyses of older patients in the DURABLE trial initiation phase, where rates of overall hypoglycemia and severe hypoglycemia were higher in older patients treated with LM75/25 compared with GL [8]. One reason for this difference may be that patients had already achieved glycemic goals in the initiation phase and were, therefore, more stable in the maintenance phase with respect to plasma glucose and insulin dose. Guidelines from the American Diabetes Association and the American Geriatric Society suggest that less stringent HbA_1C_ targets might be more appropriate for some older patients [15, 16], and the subgroup analyses of older patients in the DURABLE trial initiation phase supported these guidelines. When the subgroup analyses of older patients in the initiation phase and the current findings in the maintenance phase are considered together, the overall results might suggest that once glycemic control is attained, perhaps a less aggressive approach may not be necessary.

A potential limitation in this study is the imbalance in the number of patients taking different OAM regimens; a greater number of patients in the GL group versus the LM75/25 group were taking the SU/TZD combination at baseline. In addition, the high use of SU in this study may have influenced the overall rates of hypoglycemia and glycemic endpoints in both groups. Similar to the previous analysis of older patients in the DURABLE trial initiation phase [8], there was not an adequate comparative sample of patients not taking SU to complete a valid analysis of hypoglycemia rates and glycemic endpoints, but the possible contribution of concomitant SU use cannot be excluded.

Another limitation of this study is the smaller number of patients in the HbA_1C_ ≤6.5 % subgroup (LM75/25, *n* = 72; GL, *n* = 39), which limited the ability to draw solid conclusions from the comparison of the two treatment groups. Finally, detailed information on the use of non-diabetes-related medications, comorbidities or dietary habits was not collected in this trial. It is, therefore, difficult to determine the influence of other factors, such as underlying illness, eating habits, alcohol use or concomitant medications, on glucose control. Differences in dietary habits across the various countries could have influenced plasma glucose profiles.

## Conclusions

Despite the limitations to this study, the findings suggest that in older patients, the LM75/25 regimen resulted in longer durability of glycemic control, a greater number of patients maintaining HbA_1C_ goal, and no increase in hypoglycemia versus the GL regimen. However, this improvement in glycemic control was associated with more weight gain and modestly higher daily insulin doses. Further evaluations of meal-time insulin in older patients are warranted.
